# Lymphangiogenesis and Its Correlation with the VEGF Expression and the Sentinel Lymph Node in Cutaneous Melanomas

**DOI:** 10.1155/2014/372979

**Published:** 2014-06-25

**Authors:** Petr Buzrla, Jana Dvorackova, Oldrich Motyka

**Affiliations:** ^1^Department of Pathology, Faculty Hospital, Ostrava, Czech Republic; ^2^Institute of Pathology, School of Medicine, University of Ostrava, Ostrava, Czech Republic; ^3^Center of Nanotechnology, VSB-Technical University, Ostrava, Czech Republic

## Abstract

The aim of the study is to evaluate the density of intratumoral and peritumoral lymphatic vessels in primary cutaneous melanomas and to assess their correlation with the status of sentinel lymph nodes and the VEGF expression in tumor cells and stromal cells. A total of 40 patients were enrolled in the study: the melanomas were radically excised with the extirpation of the sentinel lymph node. The study subjects were divided into two groups: 20 cases with positive and 20 cases with negative sentinel lymph node results. The density of lymphatic vessels was evaluated by the antibody D2-40 and the VEGF expression was investigated in the semiquantitative way. The VEGF expression in melanoma cells and the stromal cells was negative to variable positive at both SLN negative and SLN positive patients in all pT stages. In the group of SLN positive patients, the density of intratumoral lymphatic vessels was low up to moderate, while it was observed to be absent, somewhere on the low level in the group of SLN negative patients. On the other side, the density of peritumoral lymphatic vessels was equally numerous at both SLN negative and SLN positive patients. The lymphatic invasion was found out at 4 SLN positive patients only. The ulceration was chiefly in the group of LN positive patients. The results show that the density of lymphangiogenesis and the intensity of the VEGF expression are considered to be an unreliable predictor of melanoma metastasis to the sentinel lymph node, but the ulceration and the lymphatic invasion can predict the potential for metastasis.

## 1. Introduction

Cutaneous melanoma is the most aggressive and malignant tumor of the skin. It is considered to be an epidemic malignant disease, as its worldwide incidence increased by 697 per cent between 1950 and 2000. The cutaneous melanoma occurs in all age groups, predominantly among the middle-aged. Both sexes are affected approximately equally, but, in Central Europe, the occurrence is higher in women. The incidence of cutaneous melanomas keeps rising up worldwide. In our population, the incidence of new cases of cutaneous melanomas rises by 2.4 per cent every year. Our statistical data have shown that the incidence of this type of cancer is 15 cases/100,000. The standard therapy primarily includes a surgical excision of the affected skin, followed by the extirpation of the sentinel lymph node. In the advanced stages of the disease with metastases, an adjuvant therapy is often indicated, including immunotherapy with interferon-*α*, radiotherapy, chemotherapy, and targeted therapy.

Lymphangiogenesis is a necessary precondition for the tumor growth and its dissemination into lymph nodes [1]. The formation of lymphatic vessels is stimulated by the proangiogenic factor VEGF (vascular endothelial growth factor), which is produced and secreted by tumor cells and stromal cells, for example, tumor-associated macrophages (TAM) and tumor-associated fibroblasts (TAF). The VEGF family comprises six structurally different related glycoproteins: VEGF-A, VEGF-B, VEGF-C, VEGF-D, VEGF-E, and PLGF (placental growth factor). These bind to three different cell membrane receptors, which belong to the family of receptor tyrosine kinases (RTK): VEGFR-1 (Flt-1), VEGFR-2 (Flk1/KDR), and VEGFR-3 (Flt-4) [2]. The result of the ligand-receptor interaction is based on the activation of tyrosine kinase domain of VEGFR which starts the intracellular signalling transduction pathways, for instance, PI3-K-AKT-mTOR and Ras-Raf-MEK-ERK [3]. The definitive outcomes of these pathways are the endothelial cell survival, mitogenesis, migration, differentiation, vascular permeability, vasodilatation, and mobilization of endothelial progenitor cells from the bone marrow to the peripheral circulation. The extensive production of VEGF continues until new vessel formation is complete [4].

Cutaneous melanomas commonly metastasize into the regional lymph nodes and their involvement in metastatic melanoma is associated with a significantly worse prognosis. The sentinel lymph node is the first lymph node on the direct lymphatic drainage path from the primary tumor. Positive sentinel node results are commonly followed by a complete dissection of the lymph nodes.

## 2. Materials and Methods

The tissues with primary cutaneous melanoma were retrospectively obtained from the archive of the Department of Pathology, University Hospital, and Ostrava University, Ostrava. The study material includes 40 invasive melanomas which were diagnosed in the last four years and were divided into two groups: 20 patients with positive sentinel lymph node results (SLN) and 20 patients with negative SLN results. There were 25 male and 15 female patients and the age varied from 30 to 82 years (the age median 61.5 years). The melanoma samples include three superficial spreading melanomas, four acrolentiginous melanomas, and 33 nodular melanomas. Their histological types, Clark's level of invasion, Breslow thickness, and ulceration are summarized in Table 1.

Immunohistochemical examination was carried out on 4 *μ*m thick serial paraffin sections, which were dewaxed in toluene and hydrated with a graded series of ethanol concentrations and water. Subsequently, antigen retrieval was obtained by using hot water bath incubation of sections in a pH 6 or 9 buffer for 20 min. Endogenous peroxidase activity was quenched by incubating the section for 10 min with 3% solution of hydrogen peroxide. Using a DAKO Autostainer device, separate subsequent sections were incubated at room temperature with D2-40 (DAKO, Denmark) and monoclonal VEGF (DAKO, Denmark) primary antibodies, respectively. Staining was performed using a technique, the Simple Stain MAX PO technique (Nichirei Biosciences Inc., Tokyo, Japan), and using 3-amino-9-ethylcarbazole (AEC) as red chromogene on the monoclonal VEGF antibody. Finally, the nuclei were counterstained with hematoxylin. VEGF staining of the hair follicle bulbs, superficial part of epidermis, sweat, and sebaceous glands were used as an internal positive control.

The immunohistochemical expression of the VEGF antibody by melanoma cells and stromal cells was semiquantitatively evaluated as follows: negative, no cells stained, low, 1–15%, moderate, 15–30%, and high, >30%.

The density of lymphatic vessels was selected in a hot spot and then counted in one high power field (40x). The distribution of these vessels was divided into intratumoral and peritumoral. The peritumoral lymphatic vessels were defined as the area confined within one microscopic field of the tumor border at 40x magnification.

## 3. Results

**Figure 1:**
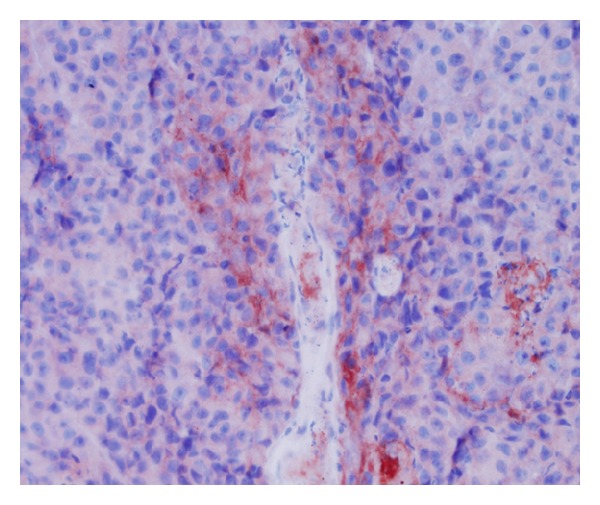
VEGF expression by melanoma cells (40x).

**Figure 2:**
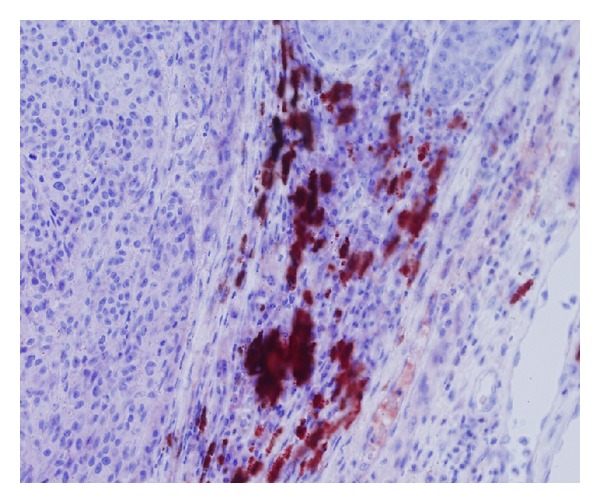
VEGF expression by stromal cells (40x).

VEGF was stained both in the melanoma cells and in the peritumoral stroma cells. We observed a very weak expression of the VEGF in the melanoma cells (Figure 1), in contrast to the stromal cells, which were stained for VEGF very strongly (Figure 2). The stroma cells are considered to be tumor-associated fibroblasts (TAF) and tumor-associated macrophages (TAM). In our study, it was predominantly TAM which were stained for VEGF. Having evaluated its expression, we found out that the melanoma cells were chiefly negative, rarely weakly up to moderately positive in all pT stages in both groups of patients, SLN positive and SLN negative, while the VEGF expression by stroma cells was mostly negative and weakly positive in patients with positive SLN. In the group of SLN negative patients, the VEGF expression by stroma cells was negative to weakly positive (Table 2).

**Figure 3:**
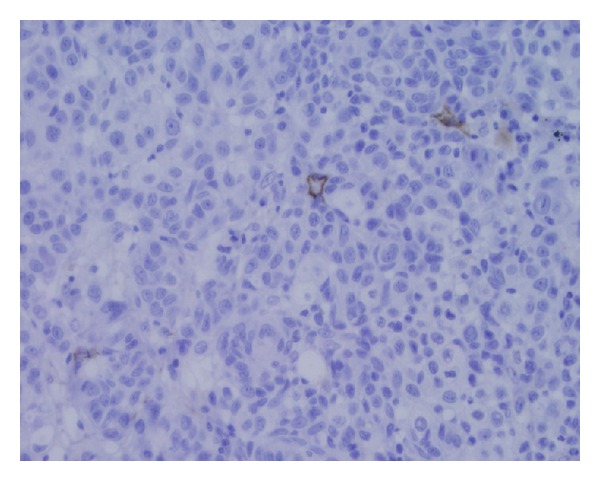
Intratumoral lymphatic vessels stained by D2-40 (40x).

**Figure 4:**
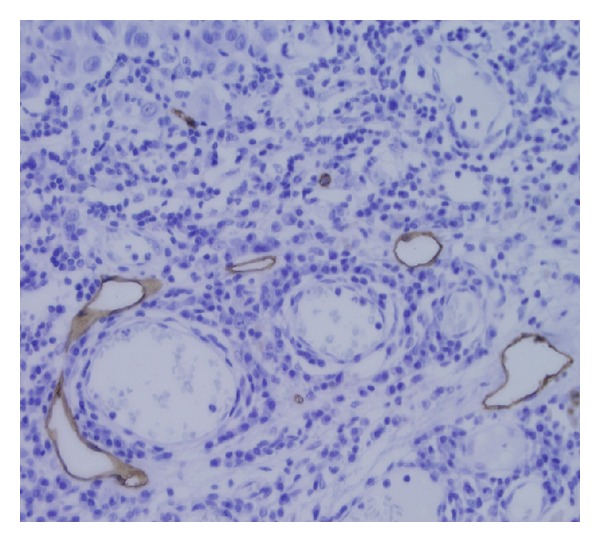
Peritumoral lymphatic vessels stained by D2-40 (40x).

We also counted the lymphatic vessels in intratumoral and peritumoral regions (Figures 3 and 4). Among the SLN positive patients, the density of intratumoral lymphatic vessels was low up to moderate. Compared to these patients, the group of SLN negative patients was associated predominantly with the absence or the low level of the density of intratumoral lymphatic vessels. Peritumoral lymphatic vessels were equally numerous in both groups of patients—SLN positive and SLN negative (Table 2).

Consequently, we detected the presence of the lymphatic invasion in 4 of 20 SLN positive patients. In the group of SLN negative patients, there was no invasion of lymphatic invasion observed. Furthermore, the ulceration was found mainly in patients with positive SLN (Table 1).

**Figure 5:**
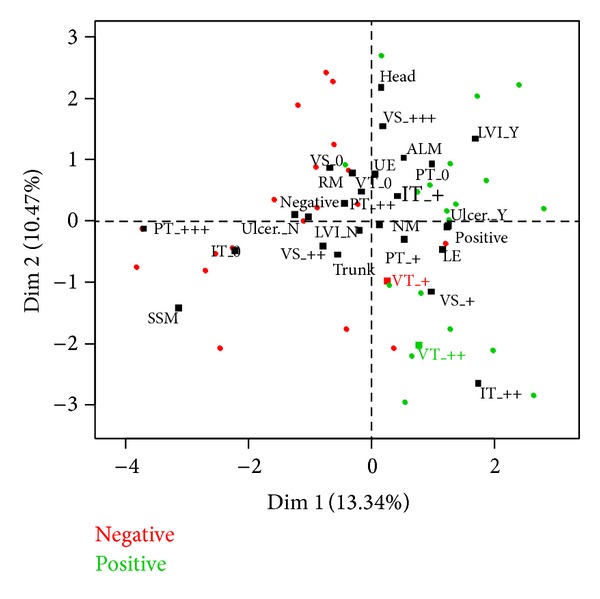


The two groups under comparison were subjected to various statistical tests to evaluate the distinctive factors (if any). Two-sample *t*-test with equal variances was used to assess the factor of age and two-sample test for equality of proportions with continuity correction for the factor of sex. There was no significant difference found in the distribution of neither of these factors so it can be assumed that the data, indeed, come from the same population; hence the groups were chosen accordingly in order to perform the other analyses (Figure 5).

The groups were found to differ in the factor of ulceration (*P* = 0.008796, two-sample test for equality of proportions with continuity correction), with ulceration itself being significantly more specific to the group of patients with negative SLN factor. Further difference found was in intratumoral density of lymphatic vessels (*P* = 0.006255, Wilcoxon rank sum test with continuity correction)—group with positive SLN factor having higher density overall. However, no significance difference was found concerning the factors of peritumoral density of lymphatic vessels, VEGF expression by tumor cells, or VEGF expression by stromal cells.

The data were also subjected to a multifactorial analysis to illustrate overall distribution of factors' effects on the two groups in question. Since the data consist of both ordinal factorial data and continuous data (Breslow), the factorial analysis on mixed data was applied; the resulting plot is presented in Figure 5. The plot shows clear distinction in the factor of SLN with both groups of the patients under study forming separate clusters; this means that this factor is indeed the crucial one. Distribution of the factors is in accordance with the conclusions derived from the statistics described earlier.

## 4. Discussion

According to the recommendation of AJCC, the patients with primary cutaneous melanoma would be managed by the radical excision of the affected skin following the extirpation of the sentinel lymph node. The histological evaluation of Clark's level of invasion, mitotic rate, ulceration, Breslow thickness of the melanoma, and the status of the sentinel lymph node leads the pathologists and the clinicians to determine the TNM stage. The sentinel lymph node as a regional one is considered to be the first node which the tumor cells invade during the drainage from the primary tumor. The presence of tumor cells in the lymph node is an indication for extirpation of other regional nonsentinel lymph nodes. The use of lymphangiogenesis as a reliable predictor of the metastasis in the lymph node has been investigated in the literature.

Our pilot study has focused on the lymphangiogenesis and its distribution and density at cutaneous melanomas and further on its correlation with the status of the sentinel lymph node and the VEGF expression by melanoma cells and by stromal cells. While other studies have used the VEGF-C as a proangiogenic factor for the formation of lymphatic vessels, we have chosen the VEGF as a family of six different vascular endothelial growth factors (VEGF-A, VEGF-B, VEGF-C, VEGF-D, VEGF-E, and PLGF), because VEGF-A also induces the active proliferation of VEGFR-2 expressing tumour-associated lymphatic vessels and tumour metastasis to a sentinel lymph node [5]. Tobler and Detmar showed that an overexpression of VEGF-A correlated with lymphangiogenesis in sentinel lymph nodes [6].

We proved a negative up to weak VEGF expression by melanoma cells in all Clark's levels of invasion in patients with positive sentinel lymph node results. Our findings are in harmony with a study published by Salven et al. [7], who described a low expression of VEGF in primary melanoma and a high expression of the same VEGF in metastatic melanomas. This statement has also been confirmed by another study of Gallego et al. [8]; however, in this study, VEGF-C was used as a prolymphatic growth factor and the authors found no difference in the intensity of VEGF-C expression according to various histological types, Clark's levels of invasion, and the Breslow thickness. In the study of Depasquale and Thompson, the VEGF expression correlates with Breslow thickness [9]. Simonetti et al. observed in their study an overexpression of VEGF in invasive melanomas only [10]. These findings of the two latter studies do not correlate with our study findings. The stromal cells around primary cutaneous melanomas also manifested a low- up-to-moderate expression of VEGF. This result corresponds with the work of Boone et al. [11]. In the group of patients with negative sentinel lymph nodes, the intensity of VEGF expression was similar to SLN positive patients. We assume that the lymphangiogenesis is stimulated mostly by VEGF and other cytokines produced by stromal cells rather than by tumor cells. However, this mechanism remains unclear.

Regarding the correlation between the density of intratumoral and peritumoral lymphatic vessels and the intensity of VEGF expression, we found no difference between them in both groups of patients with and without metastases in the sentinel lymph nodes. This finding is not supported by the studies of Dadras et al. [12] and Liu et al. [13], which state that the high expression of VEGF and the extent of lymphangiogenesis are associated with the metastases of the sentinel lymph node. Sahni et al. noted that the density of lymphatic vessels is not a reliable predictor of metastatic potential [14]. Massi et al. concluded that a high density of intratumoral lymphatic vessels was the predictor of metastasis in sentinel lymph nodes [15]. However, this conclusion was not observed in our study.

On the other hand, our investigation revealed no impact of the density of intratumoral or peritumoral lymphatic vessels on the dimensions of metastasis in sentinel lymph nodes. Furthermore, the extent of these lymphatic vessels was nearly equal in both groups of patients with positive and negative sentinel lymph nodes, but the density of such rises up in parallel with the increase of Clark's level invasion of the melanoma. This finding has also been confirmed by the work of Kashani-Sabet et al. [16].

In our set, the ulcerations of primary cutaneous melanomas were detected mostly in patients with a positive sentinel lymph node. This confirms the result of the study presented by Depasquale and Thompson [17].

The isolated cells and the micrometastases in sentinel lymph nodes were observed in melanomas in stages T1–T4, while the macrometastases were detected in T3-T4 melanomas.

The statistical analysis has found no significance difference concerning the factors of peritumoral density of lymphatic vessels, VEGF expression by tumor cells, or VEGF expression by stromal cells.

## 5. Conclusions

The VEGF expression in primary cutaneous melanomas and their stromal microenvironment is not considered to be a reliable predictor of metastasis in the sentinel lymph nodes. The density of intratumoral and peritumoral lymphatic vessels was not able to predict the potential of metastasis in the sentinel lymph node. On the other hand, the presence of the ulceration and the lymphatic invasion can predict the poor prognosis, suggesting a high probability of the metastases in regional lymph node.

The mechanisms of lymphatic invasion and progression of metastases into regional lymph nodes are hitherto poorly understood [18].

Our investigation shows that these predictive factors are not clinically useful indicators for prognostic evaluation of patients with cutaneous melanomas.

The therapeutic use of antiangiogenic drugs, for example, bevacizumab and aflibercept, has shown only a little effect, because they elicit tumour adaptation and advancement to stages of greater malignancy, with an increased invasiveness, and in some cases also increased the progression of lymphatic and distant metastases [19].

## Figures and Tables

**Table 1:** Clinical and pathological data of the study subjects.

	SLN positive (20 cases)	SLN negative (20 cases)
Median age	58.8 (33–79)	62.1 (30–82)
Sex		
Male	13 (65%)	12 (60%)
Female	7 (35%)	8 (40%)
Site		
Upper extremity	5 (25%)	4 (20%)
Lower extremity	3 (15%)	2 (10%)
Head and neck	1 (5%)	2 (10%)
Acral regions	2 (10%)	2 (10%)
Trunk	9 (45%)	10 (50%)
Thickness range	0.57–14.0 mm	0.2–12.0 mm
Clark's level		
II	1 (5%)	2 (10%)
III	9 (45%)	12 (60%)
IV	6 (30%)	4 (20%)
V	4 (20%)	2 (10%)
Ulceration		
Absent	6 (30%)	16 (80%)
Present	14 (70%)	4 (20%)
Histotype		
SSM	0	2 (10%)
NM	18 (90%)	16 (80%)
ALM	2 (10%)	2 (0%)
Lymphatic invasion		
Absent	16 (80%)	20 (100%)
Present	4 (20%)	0

**Table 2:** Summary of immunolocalization of VEGF and lymphatic vessel density.

	VEGF/t				VEGF/s				PT				IT			
	Neg.	+	++	+++	Neg.	+	++	+++	Neg.	+	++	+++	Neg.	+	++	+++
SLN+																
pT1	2	1	—	—	1	2	—	—	—	2	1	—	—	2	1	—
pT2	2	—	1	—	1	2	—	—	—	1	2	—	—	2	1	—
pT3	2	2	1	—	—	3	2	—	—	3	2	—	—	4	1	—
pT4	8	1	—	—	4	4	—	—	1	5	3	—	1	8	—	—
SLN−																
pT1	3	2	—	—	2	1	2	—	—	2	3	—	4	1	—	—
pT2	1	1	1	—	1	1	—	1	—	1	2	—	—	3	—	—
pT3	6	1	—	—	5	1	1	—	—	2	4	1	3	4	—	—
pT4	4	1	—	—	3	1	—	1	—	3	2	—	—	5	—	—

SLN: sentinel lymph node, VEGT/t: intratumoral expression, and VEGF/s: stromal expression.
